# Midwives as trainers for a neonatal clinical decision support system at four rural health facilities in eastern Uganda: a mixed-methods observational study

**DOI:** 10.1136/bmjopen-2023-081088

**Published:** 2024-11-25

**Authors:** Mary Kakuru Muhindo, Jean Armas, Moses Kamya, Elon Danziger, Joshua Bress, Theodore Ruel

**Affiliations:** 1 Infectious Diseases Research Collaboration, Kampala, Uganda; 2 Global Strategies, Albany, California, USA; 3 Division of Pediatric Infectious Diseases and Global Health, Department of Pediatrics, University of California San Francisco, San Francisco, California, USA

**Keywords:** Clinical Decision-Making, EDUCATION & TRAINING (see Medical Education & Training), Quality in health care, Health Workforce, NEONATOLOGY, Nurses

## Abstract

**Abstract:**

**Objectives:**

To evaluate acceptability and effectiveness of midwives as trainers for NoviGuide, a neonatal clinical decision support system (CDSS).

**Design:**

A 20-months, mixed-methods open cohort study.

**Settings and participants:**

Nurse-midwives at four rural health facilities in eastern Uganda.

**Methods:**

We developed a midwife-led trainer programme and instructed two midwives as NoviGuide Trainers in three 3-hour-long sessions. Trainers trained all nurse-midwives at each site in single 3-hour-long sessions. Using the Kirkpatrick model, we evaluated acceptability at level 1 for participant’s reaction and level 3 for participant’s attitudes towards the programme. We evaluated effectiveness at level 2 for newly learnt skills, and level 3 for participant’s uptake of NoviGuide and perception of newborn care practices. We used surveys and focus groups at baseline, 3 months and 6 months and viewed usage data from September 2020 through May 2022.

**Results:**

All 49 participants were female, 23 (46.9%) owned smartphones, 12 (24.5%) accessed the internet daily and 17 (34.7%) were present by study end following staff changes. All participants perceived the use of midwives as NoviGuide Trainers to be an acceptable approach to introduce NoviGuide (mean 5.9 out of 6, SD 0.37). Participants reported gaining new skills and confidence to use NoviGuide; some, in turn, trained others. Participants reported improvement in newborn care. Uptake of NoviGuide was high. Of 49 trained participants, 48 (98%) used NoviGuide. A total of 4045 assessments of newborns were made. Of these, 13.8% (558/4045) were preterm, 17.5% (709/4045) weighed under 2.5 kg and 21.1% (855/4045) had a temperature <36.5°C.

**Conclusion:**

This midwife-led programme was acceptable and led to self-reported improvement in newborn care and high uptake of NoviGuide among nurse-midwives. Task shifting CDSS expert roles to midwives could facilitate large-scale implementation. However, resources like internet coverage, reliable electricity and mobile devices should be considered in low-resource settings.

**STRENGTHS AND LIMITATIONS OF THIS STUDY**

- A multisite, mixed-methods evaluation using Kirkpatrick model (levels 1–3).
- We enrolled 49 nurse-midwives from rural health facilities and limited contact with the study team to approximate real-work implementation.
- NoviGuide Trainers had experience using NoviGuide prior to this implementation; this may have biased results towards efficacy.
- We did not evaluate the long-term impact (level 4) of the programme on newborn outcomes or health facility systems.

## Introduction

 Approximately 2.7 million newborns die every year, many from preventable or treatable conditions including prematurity, infection and birth asphyxia.^1^ Many low- and middle-income countries (LMIC) are struggling to meet the Every Newborn Action Plan target of fewer than 12 newborn deaths per 1000 live births by 2030.^2^ Health ministries urgently need approaches to upskill frontline healthcare workers to provide care to sick newborns.

The poor newborn outcomes in LMICs are due to many factors. Healthcare workers have limited skills and poor adherence to clinical guidelines; they also face large patient loads and high staff turnover, which make training difficult to implement.^3^,^5^ Clinical decision support system (CDSS) offers features that address many of these gaps in care delivery including improving fidelity to clinical guidelines and reducing medical errors.^6^,^8^ CDSS also has the potential to surmount training challenges. Incorporated into daily practice, CDSS offers the prospect of improvement that endures over time and across new and frequently changing staff. Several CDSS platforms have demonstrated efficacy in improving knowledge and confidence of frontline healthcare workers in neonatal care delivery.^9^,^12^

While CDSS is generally designed to be easy for people with smartphones and other computer experience to learn, fluency with modern technology cannot be assumed in low-resource settings. Indeed, the key challenge in scaling CDSS is that implementation requires initial training that has historically been done by information technology specialists or product designers, who are short in supply in low-income countries. Task shifting^13^ of training roles and other clinical work to lower cadre healthcare workers has been recognised as a sustainable and cost-effective method to expand access to medical care.^14^,^20^ This approach could provide a sustainable and successful implementation of CDSS. However, there have been no studies examining whether tasks traditionally done by specialists related to CDSS in low-resource settings can be task shifted to healthcare workers.

The NoviGuide Neonatal Essentials application (hereafter, NoviGuide) is a CDSS with six pathways for common neonatal encounters.^21^ These pathways include: (1) initial assessment <24 hours, (2) initial assessment >24 hours, (3) rounding, (4) spot check, (5) discharge and (6) seizure and abdominal emergency. NoviGuide contains instructional videos from Global Health Media Project^22^ depicting physical signs of common newborn conditions, breastfeeding guidance and newborn procedures. NoviGuide works both online and offline and can be deployed as a web or mobile application. It is configurable at the level of the facility to variations in equipment or protocols and its dashboards can show site-specific use. NoviGuide has been shown to improve the nurse-midwives’ knowledge and confidence to care for newborns at a rural general hospital in eastern Uganda.^6 23^

Given the limited evidence for a sustainable and scalable adoption of CDSS,^24^,^26^ our aim was to evaluate whether CDSS expert roles could be task shifted to midwives in the implementation of NoviGuide. First, we developed the NoviGuide Neonatal Essentials Trainer programme, a midwife-led training programme for the introduction of NoviGuide in health facilities. We then applied the Kirkpatrick model^27^ to evaluate: (1) the acceptability of midwives as trainers of NoviGuide among nurse-midwives caring for newborns at four rural health facilities in eastern Uganda, and (2) the effectiveness of the training programme on nurse-midwives’ uptake of NoviGuide and their perception of quality of newborn care in the four rural health facilities.

## Methods

### Study design

We conducted a 20-month, concurrent triangulation mixed-methods open cohort study from September 2020 to May 2022. We chose an open cohort study design to enrol all nurse-midwives following staff changes, capturing real-world situations with high attrition rates for NoviGuide implementation. We used Kirkpatrick’s programme evaluation model.^27^ We evaluated acceptability at level 1 (Reaction) for participant’s reaction immediately after the initial training, and at level 3 (Behaviour) for the participant’s attitude towards midwives as NoviGuide Trainers at 3 and 6 months. We evaluated effectiveness at level 2 (Learning) for participants’ newly acquired knowledge and skills to use NoviGuide, and at level 3 (Behaviour) for participant’s uptake of NoviGuide and perception of newborn care following the introduction of NoviGuide (table 1).

**Table 1:** Four evaluation levels of Kirkpatrick model and the data sources

Levels	Description	Data source	Study timeline
Level 1: Reaction			
(Acceptability)	Participants’ acceptability of midwives as NoviGuide Trainers	The Training Acceptability Rating Scale	Post-training
Level 2: Learning			
(Effectiveness)	Participants’ newly acquired knowledge and skills to use NoviGuide	Electronic Health Record End User survey Focus group discussions	3 and 6 months3 and 6 months
Level 3: Behaviour			
(Acceptability)	Participants’ attitude towards midwives as NoviGuide Trainers	Focus group discussions	3 and 6 months
(Effectiveness)	Participants’ uptake of NoviGuide Changes in newborn care practices	NoviGuide usage dataFocus group discussions	Weekly for 20 months3 and 6 months

### Study site selection and setting

We selected four government-owned health facilities located in Tororo district, eastern Uganda, where newborn care is primarily provided by nurses and midwives. The sites include Tororo General Hospital and Mulanda, Nagongera and Mukujju Health Center (HC) IVs. These health facilities serve a population of 583 400 people.^28^ Tororo General Hospital conducts approximately 400 deliveries monthly and admits nearly 100 sick newborns per month from the community, health centres, private facilities and across the Kenya-Uganda border. At the launch of the study, Tororo General Hospital had 22 midwives providing both maternal and newborn care in the labour suite, postnatal ward and a small kangaroo mother care room. Staff work 2–3 per shift on each of these units with supervisory support from two to three medical officers. They rotate to other units at least annually. The kangaroo mother care room was refurbished in September 2021 to a neonatal unit with new equipment including neonatal incubators, phototherapy, warmers and oxygen concentrators. Six staff were assigned neonatal roles to work on this unit. The three HC IVs conduct between 70 and 130 deliveries each per month and had no dedicated area for sick newborn admissions. Six to eight midwives provide newborn care in addition to maternity care including labour and delivery, postnatal care and outpatient department. Staff work 1–2 per shift with supervisory support from one medical officer.

### NoviGuide Neonatal Essentials Trainer programme

We trained two midwives as NoviGuide Trainers, tasked with instructing staff on the basic operation and troubleshooting of NoviGuide. The trainers attended three 3-hour sessions conducted at Tororo General Hospital boardroom.

There were three key elements to the training. First, trainers learnt the installation and set-up of the software onto tablets. This includes downloading the application, linking the software to a specific clinic using a 9-digit code and creating and managing user profiles.

Second, the trainers learnt how to train fellow nurse-midwives on the (1) basic function of NoviGuide, including its content and location of key functionalities such as contextual drug dosing calculators and preterm feeding widgets, (2) introduction to the use of the tablet, (3) value of NoviGuide in promoting fidelity to neonatal guidelines to reduce medical errors, (4) key safety considerations including what to do when one’s clinical judgement does not align with software information and (5) gamification features to track their use of the application.

Third, the trainers learnt how to monitor sites using the dashboards. This training includes review of dashboards, evaluation of NoviGuide adoption and address low uptake, data synchronisation and troubleshooting common technical issues.

We taught these three elements using a combination of PowerPoint presentations, role-play and support supervision. The trainers used the same PowerPoint presentations during the on-site training of nurse-midwives. In this way, the training was first modelled for the trainers.

### Enrolment and training of the study participants

All nurse-midwives at the four study sites were invited to attend an introductory session at their respective sites. Eligibility criteria included provision of newborn care at the study sites, more than 18 years of age, having an active practising licence to practise and willingness to participate in the study. We obtained written informed consent before participation. We conducted recruitment first at Tororo General Hospital in September 2020 and at Mulanda, Nagongera and Mukujju HC IVs in March, April and May 2021, respectively. The delay was due to COVID-19 pandemic travel restrictions.

Following recruitment, NoviGuide Trainers travelled to each site and trained participants for 3 hours. The trainers used the PowerPoint presentations of the NoviGuide Neonatal Essentials Trainer programme. The trainers then provided technical support for NoviGuide use and addressed any technical problems daily during the first 2 weeks and monthly thereafter. As an open cohort study, the trainers also trained newly recruited participants following staff changes.

### Introduction of NoviGuide at the health facilities

The NoviGuide Trainers introduced NoviGuide at the four study sites, adding each participant into the application as a user with a distinct password. Each site received two to four tablets (Amazon Fire HD 8 tablet) loaded with NoviGuide. The number of tablets depended on the number of staff per shift. With two to three staff per shift, the general hospital received four tablets, while the HC IVs each received two tablets because they have one to two staff per shift. Tablets were stored in lockable wooden cabinets located in the midwives’ office, neonatal unit or labour suite.

The trainers identified a key contact person (NoviNurse) at each site who kept the study team informed about any challenges encountered. The NoviNurse assisted with data synchronisation by intermittently connecting the tablets to WiFi provided by a MiFi modem (Airtel 4G modem model MF927U).

### Data collection

At baseline, participants completed a questionnaire that included demographic information (age and sex), years of clinical experience, role (nurse or midwife), health facility, devices personally owned and access to internet.

Immediately after the training, participants completed a survey adapted from the Training Acceptability Rating Scale evaluating acceptability (Kirkpatrick level 1—Reaction). The first section^29^ consists of six statements assessing general acceptability, appropriateness and perceived effectiveness, negative side effects, consistency and social validity of midwives as NoviGuide Trainers. The participants rated each of the statements on a 6-point Likert scale indicating their degree of agreement or disagreement with responses 1–6, where 1 represents ‘Strongly disagree’ and 6 represents ‘Strongly agree’. The second section^30^ consists of nine statements assessing the participants’ perception about the training process and competence of Trainers. The participants rated each of the statements on a 4-point scale from 1 to 4 where 1 represents ‘Not at all’ and 4 ‘A great deal’.

At 3 and 6 months, participants completed another survey adapted from the Electronic Health Record End User survey and participated in focus group discussions for evaluation of both effectiveness and acceptability (Kirkpatrick level 2—Learning and level 3—Behaviour). We chose 3 and 6 months as these timepoints were close enough to the initial training for participants to remember their perception about the programme. The survey consists of 11 statements, including: ‘I received adequate training from the Trainers on how to use NoviGuide’, ‘My questions about NoviGuide were sufficiently answered by the Trainers’ and ‘The Trainers were able to provide technical support on NoviGuide when I needed it’. The respondents indicated their degree of agreement or disagreement on a 5-point Likert scale, where 1 represents ‘strongly disagree’ and 5 ‘strongly agree’. In total, we conducted 10 focus group discussions; four at the general hospital because of the large number of participants and two at each of the three HC IVs. Seven to eleven participants attended each discussion. Two female members of the study team guided the discussions. One moderated the discussions using the following questions: (a) Share your experience of caring for newborns before and after the introduction of NoviGuide, and (b) Describe your attitude towards midwives as NoviGuide Trainers. The other managed the audio recording. Each focus group opened with a statement explaining the purpose of the discussion and an assurance of confidentiality. All the interviews were conducted in English, audio recorded and lasted approximately 1 hour. The focus group data were transcribed verbatim, labelled with a unique number and kept on a password-protected computer.

At least once every week for 20 months, NoviNurses connected the tablets to WiFi by switching on modems to transfer NoviGuide usage data to a secure cloud-based database. We viewed the usage data from the site dashboards for the participant’s uptake of NoviGuide to further evaluate effectiveness (Kirkpatrick level 3—Behaviour).

### Data analysis

We used descriptive statistics to analyse the participant’s baseline characteristics and NoviGuide uptake. For the Training Acceptability Rating Scale 1 and 2, we determined the mean scores and SD of each of the statements. For each of the Electronic Health Record End User survey statements, we calculated the median scores and IQRs. We then used a two-tailed Wilcoxon signed-rank test to compare the median scores at 3 and 6 months. To determine the magnitude of the differences, we calculated effect size using Cohen’s d. A positive effect size indicated an increase in the mean score while a negative effect size indicated a decrease. We considered effect sizes of 0.2 to <0.5 as small, 0.5 to <0.8 as medium and 0.8 and above as large.^31^ To assess uptake, we determined the proportion of trained participants who used NoviGuide following the training. We also captured the total number of newborn assessments entered into NoviGuide by each participant, expressing the results in a figure and determined the total and range of assessments per site. We analysed all quantitative data using Stata V.16 (StataCorp, College Station, Texas) setting the CI at 95% and considered p value <0.05 significant.

Qualitatively, we employed thematic analysis^32^ using Qualitative Data Analysis Miner Lite V.2.0.9 (Provalis Research, Montreal, Quebec, Canada). MKM cleaned the data by reading each transcript while listening to the original recording. Then, MKM and JA analysed the data. During the coding meetings, they developed subthemes emerging from the codes, further categorising these subthemes into three overarching themes: one theme in level 2 (Learning)—Newly gained knowledge and skills, and two themes in level 3 (Behaviour)—Changes in newborn care practices and Attitude towards midwives as trainers. In total, 10 subthemes emerged. For each subtheme, we included key illustrative quotes and examined for similarities and differences across study sites. The whole study team approved the finalised categorisation of the subthemes.

We concurrently triangulated the quantitative and qualitative data by assessing focus group discussion data for content areas that explained or contradicted survey data and NoviGuide usage data. We used Strengthening the Reporting of Observational Studies in Epidemiology checklist^33^ for cohort studies during the preparation of this manuscript.

### Patient and public involvement

Neither patients nor the general public were involved in the design or management of the study.

## Results

### Participant characteristics

We screened 49 female nurse-midwives and enrolled them all with a mean age of 34 (range: 24–56) years. None declined to participate in the study. Of 49 participants, 26 (53.1%) worked at Tororo General Hospital and 8 (16.3%), 8 (16.3%) and 7 (14.3%) at Mulanda, Mukujju and Nagongera HC IVs, respectively. We first enrolled participants in September 2020 from Tororo General Hospital (13/26) before adding Mulanda, Nagongera and Mukujju HC IVs in March, April and May 2021, respectively. This was an open cohort with new participants enrolled during the course of the study following staff changes. Majority (46/49 (94%)) were midwives and only 3/49 (6%) were nurses, 23/49 (46.9%) owned smartphones but only 12/49 (24.5%) reported accessing the internet daily. Of 49 participants, 21 (42.7%) had work experience of 3–10 years, 12 (24.5%) had worked for 11–20 years, 11 (22.5%) for 0–2 years and only 5 (10.2%) for more than 21 years.

Of 49 enrolled participants, only 17 (35%) remained in the study at the time of closure because of staff changes, including: (a) transfer to other wards within or to other health facilities by the district administration, and (b) refurbishment of the neonatal unit at Tororo General Hospital in September 2021, where only six participants were assigned to care for newborns. The rest were given non-neonatal assignments.

### Kirkpatrick level 1: Reaction

Immediately following the initial training, all participants reported high acceptability of midwives as trainers of NoviGuide (mean 5.9, SD 0.37) (table 2). They perceived the use of midwives as NoviGuide Trainers an appropriate approach that would result in increased interest in NoviGuide among staff. The participants perceived the training by midwives sufficient for them to develop the skills needed to use NoviGuide during their care of newborns (mean 3.88, SD 0.33). The participants felt confident to use NoviGuide (mean 3.80, SD 0.41). The trainers were perceived as competent, motivating and able to relate well with the participants during the training sessions.

**Table 2:** The Training Acceptability Rating Scale (Kirkpatrick evaluation level 1—Reaction)

Questions	Mean score (SD)
The Training Acceptability Rating Scale 1 (maximum score of 6)*	
1. General acceptability: Midwife trainers would be appropriate for other staff at other hospitals and clinics.	5.9 (0.37)
2. Effectiveness: This training approach will be beneficial for the staff.	5.94 (0.24)
3. Negative side effects: This training approach will result in decreased interest in using NoviGuide.	1.33 (1.14)
4. Appropriateness: Most staff would not accept midwife trainers as an appropriate approach to learn how to use NoviGuide.	1.78 (1.65)
5. Consistency: The training was consistent with common sense and good practice in helping staff learn to use the NoviGuide in the care for newborns.	5.84 (0.75)
6. Social validity: In an overall general sense, most staff would approve of midwives as NoviGuide Trainers.	5.76 (1.01)
The Training Acceptability Rating Scale 2 (maximum score of 4)†	
7. Did the training improve your understanding of NoviGuide?	3.76 (0.48)
8. Did the training help you develop skills you need to use NoviGuide, that is, you feel comfortable using the tablet and the NoviGuide software?	3.88 (0.33)
9. Has the training made you feel confident about using NoviGuide?	3.80 (0.41)
10. Do you expect to make use of what you have learnt in the training when you use NoviGuide?	3.86 (0.35)
11. How competent were the midwife trainers?	3.86 (0.35)
12. In an overall general sense, how satisfied are you with the training?	3.71 (0.46)
13. Did the training set out to cover the topics it set out to cover?	3.65 (0.56)
14. Did the midwife trainers relate to the group effectively?	3.96 (0.20)
15. Were the midwife trainers motivating?	3.92 (0.28)

* 1: Strongly disagree, 2: Moderately disagree, 3: Slightly disagree, 4: Slightly agree, 5: Moderately agree, 6: Strongly agree.

† 1: Not at all, 2: A little, 3: Quite a lot, 4: A great deal.

### Kirkpatrick level 2: Learning

Three subthemes emerged concerning the participants’ newly gained knowledge and skills, namely: (1) learnt how to use NoviGuide, (2) gained technology skills and (3) colleagues learnt and helped others (table 3). Across all study sites, participants reported that the training was sufficient for them to start using NoviGuide. They discussed that NoviGuide was easy to learn because of the understandable language and guidance provided by the software. Some of the participants, especially those who did not own smartphones, reported gaining new skills in using technology. In addition to the Trainer’s support, participants also reported receiving support from their colleagues, especially during weekend and night shifts. Support sought included: (a) how to navigate the tablet, (b) waking an unresponsive tablet and (c) training colleagues who were away during the initial training.

**Table 3:** Focus group discussion themes and key illustrative quotes (Kirkpatrick evaluation levels 2 and 3)

Theme	Subtheme	Key illustrative quotes
**Kirkpatrick evaluation level 2—Learning**		
Newly gained knowledge and skills	Learnt how to use NoviGuide	‘The training was okay. We were able to use NoviGuide.’ (NAG-03)‘…it did not take us a lot. Within one week, we had caught up and we were doing perfectly…’ (MUK-03)‘To me, NoviGuide uses the language we understand. There are no serious terminologies in this NoviGuide and sometimes even when you mess up with something, it tells you…’ (MUL-01)
	Gained technology skills	‘At the very beginning, I faced a challenge because the technology, I was not used to it. I was used to analogue. Everything with a pen. When I continued to practice, I felt it was the easiest way of managing these children. At least I felt my technology was also improving a bit. I have moved a step away from analogue…’ (MUL-05)
	Colleagues learnt and helped others	‘… sometimes you can get stuck somewhere and consult a colleague. One time, I remained stuck on the treatment but I didn’t know where to go. So, I consulted a colleague and she directed me.’ (MUL-03)‘…we had a colleague that we trained later…so we had to guide her on what to do… we made sure that the first five babies that we had, she was the one to start NoviGuiding. So, we were like supervising and telling her what to do. So that’s how she was able to catch-up very fast and to use NoviGuide up to now.’ (MUK-07)‘When I see sister, she asks me, ‘What is the problem?’ And if you tell her, maybe the tablet is down… she can say, ‘Try this.’ So that is how sometimes we do it if our Trainers are not around.’ (TOR-09)
**Kirkpatrick evaluation level 3—Behaviour**		
Changes in newborn care practices	Confidence to care for newborns	‘… those days, when that baby is brought, you even begin thinking… what am I going to do to this baby? But now…we have the confidence. These babies are received in time and given treatment in time.’ (TOR-15)‘Before NoviGuide, I would get worried whenever I received a sick baby. I would call my seniors and eventually we would refer the baby. But ever since NoviGuide came, am comfortable.’ (MUK-05)‘…you would call a doctor and he or she may take maybe two hours without arriving to save my baby. But now, meanwhile, as we wait for them, you are at least able to identify what you're supposed to do… sometimes they tell you to refer the baby. It’s already late, so babies would die.’ (TOR-05)‘[NoviGuide] has also simplified our work…we could struggle to first of all look for our phones to calculate the doses. But with NoviGuide, you just pick the tablet…it will give you the exact treatment…’ (MUL-03)
	Newborn care knowledge	‘NoviGuide has taught me a lot, I used to overdose the children. I didn’t know what to do…’ (NAG-02)‘I get set and I know which baby will need NoviGuide without fail, before even this baby is born. And when the baby is born, I will know NoviGuide will tell me what to do.’ (TOR-11)‘Before, we could give syrups…’ (MUK-01)’…we used to hear about phenobarbitone and originally, we could even dissolve the tablet at school but we didn’t know the right quantity. They could tell you quarter of the full tablet. But now we came to know the full doses.’ (MUK-02)‘NoviGuide has helped me to give me warning signs. For example, preterm, once you input the temperature, it [NoviGuide] will warn you whether the baby needs extra warmth. When the glucose levels are low, it will warn you. I didn’t know the normal ranges but it [NoviGuide] guides you. And when it [the newborn] needs fluids, it [NoviGuide] will tell you that it [the newborn] needs the fluids.’ (MUK-03)
	Teamwork	‘…when there was a challenge, we could come together as a team and ask each other, now here, what can I do? So as a facility, we have been having good teamwork and there was nobody who was left behind.’ (MUL-05)‘NoviGuide involves togetherness. So, like, I can come in the morning, and the baby has been delivered by a night nurse. And then I NoviGuide the baby. So, I have to handover to the next person, maybe for rounding off. So, it has helped us in this teamwork and togetherness. And that is also really good for the management of babies.’ (TOR-05)’…I have a baby, maybe I have tried to cannulate and I failed, most times I call my colleagues. There are people who may be better than you. So, we always put our hands together.’ (TOR-15)‘… there were so many things I would doubt. Can this baby live? And after this and that treatment, we were finding life easy…two or three of us come together and work was fine.’ (NAG-05)
Attitude towards midwives as trainers	Confidence in a fellow midwife	‘To me, I appreciate a fellow midwife to train… somebody from a different place gives you that fear to ask some questions and I feel being a fellow midwife training you, you’re free and you can ask anything.’ (TOR 17)‘…We have confidence in them…’ (TOR-13)’We are used to them so we interact like colleagues. There is no fear…than bringing people from somewhere else.’ (MUK-08)
	Accessibility	‘…they are easily accessible.’ (TOR-17)‘…This is a person I’m always with. In case I’m stuck with anything, I can easily consult her.’ (TOR- 03)‘…even in the night, we work with them.’ (TOR-11)‘…and they are just a call away in case of anything.’ (MUK-07)
	Easy communication	‘To me I see its good… when a midwife and a midwife talk to each other, at least there are simple words that they understand. But now…a doctor talking to a midwife, I will start imagining which question is the doctor going to ask. But if it’s a midwife with a midwife…they all speak the same language.’ (MUL-04)‘…you can talk the same accent… sometimes we have experience when we get some people to train us and you would say pardon and again pardon, then the person will get a little irritated so will not explain well. They [Trainers] explained clearly…’ (NAG-05)‘…even when I couldn’t understand in English they could translate in our local language.’ (MUK-01).
	Midwives knowledgeable about newborn care challenges	‘…they [Trainers] know what is on the ground and what to be done. Because somebody from out will not know what happens, what we go through…’ (TOR-22)‘… they [Trainers] knew very well…the challenges we are facing, what we had and didn’t have, and they helped us to work within our limited resources to be able to help these babies survive.’ (TOR-11)‘They are able to explain to us in the simplest way and we feel our people are being empowered to work with us. They are knowledgeable, hard working. So I feel they can roll this [NoviGuide] across the country.’ (MUL-07)

MUKMukujju Health Center IVMULMulanda Health Center IVNAGNagongera Health Center IVTORTororo General Hospital

The focus group data aligned with the survey data (table 4). The participants consistently strongly agreed at 3 and 6 months (median 5, IQR 5–5) that they received adequate training from the NoviGuide Trainers on how to use NoviGuide (z=−1.65, d=−0.26); that their questions about NoviGuide were sufficiently answered (z=0.58, d=0.95); and that the Trainers provided technical support when needed (z=1.07, d=0.14). However, these were all not statistically significant.

**Table 4:** Electronic Health Record End User survey (Kirkpatrick evaluation level 2—Learning)

Survey statements (maximum score of 5)*[Table-fn T4_FN1]	3 months(n=38)	6 months(n=36)	Comparing scores at 3 and 6 months		
			Wilcoxon signed-rank test		Effect size
	Median score(IQR)		*z*	P value	Cohen’s d (95% CI)
1. I received adequate training from the midwife trainers on how to use NoviGuide.	5 (5–5)	5 (5–5)	−1.65	0.10	−0.26 (−0.72 to 0.2)
2. My questions about use of NoviGuide were sufficiently answered by the Trainers.	5 (5–5)	5 (5–5)	0.58	0.56	0.95 (−0.36 to 0.55)
3. The Trainers provided technical support on NoviGuide when I needed it.	5 (5–5)	5 (5–5)	1.07	0.29	0.14 (−0.32 to 0.59)
4. I am satisfied with the support I have received from the Trainers to use NoviGuide.	5 (5–5)	5 (5–5)	0	1.00	−0.05 (−0.50 to 0.41)
5. There were many times when NoviGuide was not working.	2 (1–4)	1 (1–4)	1.46	0.14	0.23 (−0.23 to 0.69)
6. When NoviGuide is not working, I know what to do.	5 (4–5)	5 (4–5)	0.51	0.61	−0.02 (−0.48 to 0.44)
7. The NoviGuide screens respond to my actions instantly.	5 (5–5)	5 (4–5)	1.71	0.087	0.36 (−0.10 to 0.82)
8. Our clinic has adequate tablets to use NoviGuide.	5 (4–5)	5 (4.5–5)	−0.88	0.38	−0.24 (−0.10 to 0.22)
9. The project plan was adequately communicated to us when NoviGuide was introduced.	5 (5–5)	5 (5–5)	−0.82	0.41	−0.74 (−0.53 to 0.38)
10. The administration of our facility was supportive during the introduction of NoviGuide.	5 (5–5)	5 (5–5)	−0.07	0.95	−0.24 (−0.48 to 0.43)
11. Adequate resources were committed to the introduction of NoviGuide.	4 (4–5)	5 (4–5)	−1.15	0.25	−0.17 (−0.63 to 0.28)

* 1: Strongly disagree, 2: Somewhat disagree, 3: Neutral or no opinion, 4: Somewhat agree, 5: Strongly agree.

### Kirkpatrick level 3: Behaviour

From the focus group discussion data, we identified two main themes related to behaviour: (a) changes in newborn care practice and (b) attitude towards midwives as trainers (table 3).

From the theme Changes in newborn care practices, three subthemes emerged: (1) improved confidence to care for newborns, (2) improved newborn care knowledge and (3) improved teamwork. All participants across the study sites reported improved confidence to care for newborns after the introduction of NoviGuide. They reported the ability to initiate treatment for sick newborns that would otherwise have waited for a doctor or clinical officer or have been referred to a higher facility without any care. NoviGuide ‘brought us from darkness to some light in the management of the children’ (MUL-05), specifically in: (a) drug choices, (b) drug mixing and reconstitution and (c) decisions on what to do for a sick newborn. The participants attributed their newly gained knowledge to NoviGuide’s unique features like the prompts, automated drug and fluid calculations and the drug mixing instructions, which simplified their work compared with using phone calculators or memory recall.

From the theme Attitude towards midwives as trainers, four subthemes emerged: (1) confidence in a fellow midwife, (2) accessibility, (3) easy communication and (4) midwives knowledgeable about newborn care challenges. Participants across all the study sites expressed their confidence in fellow midwives as NoviGuide Trainers. Only one participant from Tororo General Hospital expressed a desire to be trained by technology experts. They all agreed that midwives were easily accessible for consultations using simple terminologies and the local language to explain certain concepts. The trainers were viewed as knowledgeable about newborn care challenges faced by rural facilities and therefore well suited to appropriately use that information during the training. Participants perceived the use of midwives as trainers to be empowering and an option for introducing NoviGuide to other sites.

**Figure 1:**
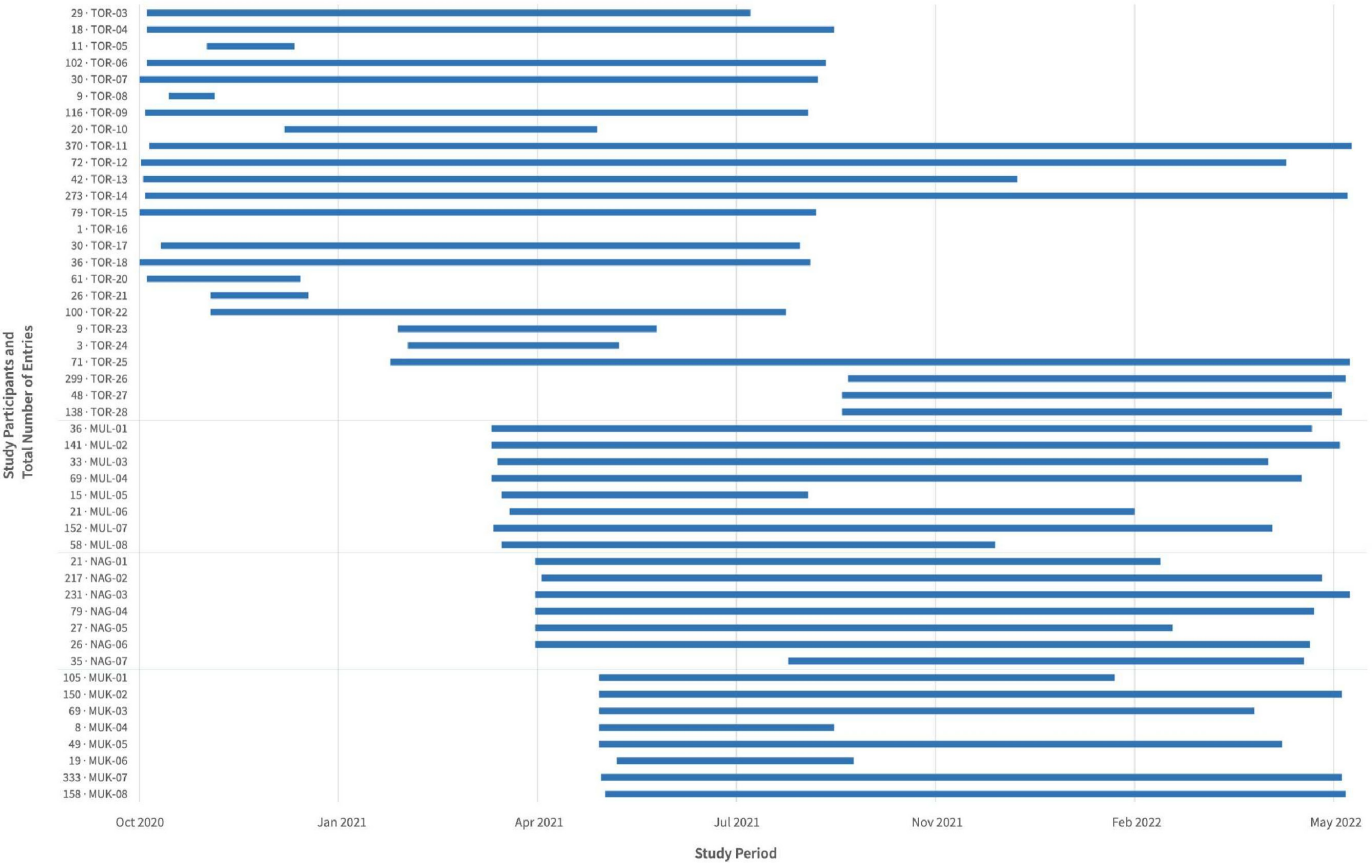
Uptake of NoviGuide by each study participant throughout the study period, evaluating the effectiveness of the training programme at Kirkpatrick level 3—Behaviour. MUK, Mukujju Health Center IV; MUL, Mulanda Health Center IV; NAG, Nagongera Health Center IV; TOR, Tororo General Hospital.

The usage data aligned with the focus group discussion data about the participant’s use of NoviGuide. Of 49 trained participants, 48 (98%) used NoviGuide following the training (figure 1). One participant from the general hospital was transferred to another facility immediately after the training. In 20 months, from September 2020 to May 2022, a total of 4045 assessments of newborns were entered into NoviGuide. The use of NoviGuide varied among participants from different sites. Of the 4045 assessments, participants from Tororo General Hospital made 1993 (49%) assessments (range: 1–370), while participants from Mulanda, Mukujju and Nagongera HC IVs made 525 (13%) assessments (range: 15–152), 891 (22%) assessments (range: 8–333) and 636 (16%) assessments (range: 21–131), respectively.

The participants used NoviGuide for varying newborn conditions. Of all the assessments made into NoviGuide, 13.8% (558/4045) were for preterm newborns, 17.5% (709/4045) for newborns weighing under 2.5 kg and 21.1% (855/4045) had a temperature less than 36.5°C.

In addition to NoviGuide use during the admission of newborns to the hospital, participants used NoviGuide for rounding (12.2% (492/4045)) and during discharge (9.3% (376/4045)), where they used a checklist to assess readiness for discharge. The participants also viewed instructional videos within NoviGuide. These videos, as discussed during the focus groups, were used especially for first time mothers and as a quick reminder of clinical skills. Of the 44 participants whose use of NoviGuide videos was assessed, 25 (56.8%) watched breastfeeding videos, 10 (22.7%) a discharge video and 9 (20.5%) a video on danger signs. For reminders on clinical skills, 22 (50%) watched a resuscitation video, 16 (36.4%) an intravenous insertion video and 12 (27.3%) a video on how to insert a nasogastric tube.

## Discussion

In our study, the participants revealed a high degree of acceptance of midwives as NoviGuide Trainers. The midwife-led training programme resulted in high uptake of NoviGuide among participants and perceived improvement in newborn care practices.

Task shifting has been shown as a cost-effective strategy for addressing staff shortages in the provision of high-quality care for chronic medical conditions.^34^,^36^ Most studies on neonatal CDSS in Africa, including our previous research, evaluated the acceptability and feasibility of individual products.^6 37 38^ This study contributes to the limited evidence essential for the sustainable scale-up of CDSS in similar settings. However, CDSS can also be considered a health system strengthening intervention requiring changes at the individual, facility and health system levels.^26 39 40^ Successful adoption of complex interventions requires workflow adjustments at different levels of the health systems while tackling drug shortages, frequent staff transfers, heavy workload and staff training and support. Additional resources are needed for CDSS implementation including internet coverage, reliable electricity and mobile devices which may not be easily available in rural settings.

Characteristics of the CDSS should also be considered for adoption success.^9^ Our findings of improved confidence and newborn care practices are consistent with other studies on CDSS.^7^,^10^ However, contrary to other studies^39 41^ where poor computer skills are reported as a barrier to implementation of CDSS in African settings, lack of prior exposure to smartphone use was not a hindrance to NoviGuide use in our study. Uptake of the software was very high. We attribute this to the usability and unique functionalities of NoviGuide. NoviGuide uses simple terminology and provides easy-to-follow guidance at the point of care. To further improve care, usage data could potentially be used to support quality improvement initiatives and staff supervision. Further research is therefore required to evaluate how site dashboards could be integrated into routine organisational systems.

Our results also aligned with studies^42^,^44^ where midwives effectively took on additional roles as trainers for facility-based interventions. However, it is important to put into consideration the implications that task shifting has on other roles of the midwife trainers. This is because trainers are removed from clinical care to prepare for and provide training. A cost-effectiveness evaluation is needed to compare this approach to conventional methods.

Our study has several limitations. To avoid selection bias, we enrolled all nurses and midwives caring for newborns at the respective health facilities. However, two trainers in our programme had previous experience with NoviGuide; this likely contributed to the effectiveness of our training programme. New trainers would likely take additional time for training. However, the idea of using users as trainers is an appealing strategy for scaling CDSS. Our work describes a task-shifting approach from CDSS experts to midwives. We did not formally evaluate the implications that task shifting had displacing other administrative roles of the midwives. While the universally positive opinions from participants about the programme are encouraging, it raises concerns about reporting bias; it is possible that participants felt obligated to give their supervisors positive reviews as trainers. However, the CDSS data suggest that participants sought to use the CDSS even outside of the presence of the trainers. One site had recently undergone refurbishment of their neonatal unit. The perception of better care resulting from new resources at that facility could have positively biased participants’ perception of improved quality of newborn care. We evaluated outcomes at Kirkpatrick levels 1–3. Therefore, further research at level 4 is required for long-term impact of the training programme on newborn outcomes and organisational systems.

## Conclusion

The use of midwives as NoviGuide Trainers was acceptable in the introduction of a complex neonatal CDSS among nurse-midwives at four rural health facilities in eastern Uganda. The trained midwives provided technical support and NoviGuide troubleshooting. This support resulted in high uptake of NoviGuide among nurse-midwives and improved confidence and self-reported improvements in neonatal care timeliness, accuracy and team communication. Task shifting information technology roles to midwives could play a key role in the scale-up of CDSS. However, resources including internet coverage, reliable electricity and mobile devices should be considered for sustainable scale-up in low-resource settings. Further research is also required on the cost-effectiveness and long-term impact on newborn outcomes and organisational systems.

## Data Availability

Data are available upon reasonable request.
