# Supplementary material for: Midwives as trainers for a neonatal clinical decision support system at four rural health facilities in eastern Uganda: a mixed-methods observational study
Source: BMJ Open. 2024 Nov 25;14(11):e081088. doi: 10.1136/bmjopen-2023-081088 (PMC11590793; doi:10.1136/bmjopen-2023-081088)
Supplement: Supplementary file 1 [file bmjopen-2023-081088.reviewer_comments.pdf]

PEER REVIEW HISTORY

BMJ Open publishes all reviews undertaken for accepted manuscripts. Reviewers are asked to complete a checklist review form and are provided with free text boxes to elaborate on their assessment. These free text comments are reproduced below.

ARTICLE DETAILS

Title (Provisional)

Midwives as trainers for a neonatal clinical decision support system at four rural health facilities in eastern Uganda: A mixed methods observational study

Authors

Muhindo, Mary Kakuru; Armas, Jean; Kamya, Moses; Danziger, Elon; Bress, Joshua; Ruel, Theodore

---

VERSION 1 - REVIEW

**Reviewer**	**1**
**Name**	**Abt, Maryline**
**Affiliation**	**University of Applied Sciences Western Switzerland La Source Institute and School of Nursing**
**Date**	**09-Nov-2023**
**COI**	**I declare that I see no conflict of interest**

The problem needs to be described in more detail. In fact, the background should describe what midwives and nurses lack in terms of assessment. How is this gap filled by the use of the application? Are these staff poorly trained to welcome newborns?

Distinguish between the two objectives. For greater clarity, we need to distinguish between 1) the acceptability of using the application and 2) the effectiveness of the fact that it is carried out by midwives. For each of these objectives, the outcomes selected to achieve them should be linked.

Explain why you carried out 11 focus groups?

You'd better explain why the 3-month and 6-month measurements answer the question. Explain why you made this choice. It's not clear, and with such a high attrition rate, how were newcomers trained?

The discussion is more like an advertising brochure than a reflection on the results. Moreover, it presents these results again without discussing them. These results should be put in perspective with what the literature says on the subject: what the literature says

about the influence of making the training by healthcare professionals? in the literature, etc.?

**Reviewer**	**2**
**Name**	**Ahmed, Wisal**
**Affiliation**	**University of Washington, Global Health Department**
**Date**	**19-Nov-2023**
**COI**	**I have no competing interests to declare.**

The authors evaluated the acceptability and effectiveness of midwives as trainers of a digital clinical decision-making application amongst midwives/nurses working in a selected hospital and health centers in Uganda. The study contributes to literature on shifting tasks from information technologists to health workers at primary health care.

General comments:

1. Study Site selection and setting lines Page 5 27 – 43.

a. It will be useful to reader to describe study population more. What was the number of midwives in hospital and health center level? How long do they stay in their post in each facility? What is the rotation system to work in maternity/neonatal units specifically for hospital.

b. The neonatal room refurbishment in September is an important detail with regards to improving quality of care for neonates in general and would impact results. This is missing in discussion and would be important to indicate if there were significant differences between 3 and 6 months for hospital level compared to health centers?

2. The use of the term “low resource settings” is too general for recommendations or application of this approach and suggest avoiding its use as a generality and qualifying to study/specific settings in abstract, discussion and conclusion section. The ownership of tablet ownership, internet coverage and electricity reliability are important factors that need to be considered in “cost effectiveness” or feasibility to make an overarching recommendation for low resource settings.

3. METHODOLOGY:

a. MAJOR COMMENT: Kirkpatrick’s four levels are 1 (reaction), 2 (learning objectives met), 3 (behavior change i.e., attitudinal or translation of learning into practice) and 4 (impact on organization, quality and user of service). Currently level 4 measure is change of behavior – which is uptake of NoviGuide and should be categorized as Level 1.

b. MAJOR COMMENT: Level 4 measure i.e. the uptake of use of CDSS is dependent on other factors (internet, smartphone) besides training. Not controlling for these factors makes it difficult to attribute training effectiveness. This finding and methodology to assess it is quite weak and best to limit evaluation to current levels 1, 2 and 3.

c. What was the eligibility criterion for enrolling midwives in the facility? For instance, were all the midwives and nurses in the hospital invited to participate. What about ownership of smartphones?

d. There was mention in open cohort in result section that was not covered in methods. Why was this approach used.

e. Page 6 line 52. Can you explain how tablets were distributed? What was the ratio, tablet per how many nurses/midwives. Similarly, Can authors explain how internet presence/absence affected use of application. About a quarter accessed internet and not clear to reader what this means, does this mean that they did not use the app?

4. What does “sustained use” mean? (line 51 in Abstract)., best to be specific that it covered three months period.

5. Am curious to learn, why trainees’ skills were not built on monitoring dashboards within their facilities to empower them to improve quality and not depend on trainers.

6. Organization of manuscript – ethical considerations, funding, acknowledgement, author contribution and conflict of interest should be placed before references not in method section

7. Patient and public involvement lines 17 -29: The content would need to be revised. District health officer and retired midwife are professionals and do not constitute the public or patient populations. Authors would need to explain whether involvement of patients or public was appropriate or not for their study.

8. Result section

a. GENERAL MAJOR COMMENT: The results are biased to only positive findings which is strange given the complexity/new approach used and Table 2 indicate some negative findings on acceptability and Table 3 in use that would need to be reflected in result section and in discussion. Recommend a balanced reporting of positive and negative findings.

b. It would be nice to see more demographic details on study population such as the mean (range) time working within maternity/neonatal units, professional years of work in general

c. Results on drug dosing are hard to interpret. What is the intention of communicating these results. If it is to describe the commonest areas used in NoviGuide, suggest to enter the findings in a table indicating what aspect of dosage was often accessed, admissions, discharge etc. disaggregating by health facility type (hospital vs health center level). What does the percentage mean? What is the denominator?

9. Discussion

a. The first two paragraphs is a repeat of study findings and could be summarized into two lines.

b. Recommend section to be written to focus on result interpretation, for instance, what needs to be studied further.

i. For instance, questions that remain unanswered is the effectiveness of midwives trainers on the quality of neonatal care (level 4) or long term impact on uptake given that assessment was 3 – 6 months post intervention which is short and Hawthorne effect/bias could have been high

ii. What does this scale up of this approach at subnational or national level and its implications in selection and training of trainers? Would more research need to be done on how monitoring could be integrated within routine supervision or more investigation made on time spent in troubleshooting and the appropriate number of trainers to cover health facilities

c. What does turnover of staff mean, mobile ownership, internet usage, how could this affect overall effectiveness? Can this be controlled in future studies? Have other studies looked at this?

VERSION 1 - AUTHOR RESPONSE

Reviewer #1

1. The problem needs to be described in more detail. In fact, the background should describe what midwives and nurses lack in terms of assessment. How is this gap filled by the use of the application? Are these staff poorly trained to welcome newborns?

   Our response: We appreciate the reviewer's comment. We have clearly defined the problem in the 'Introduction' section as follows:

   > *The poor newborn outcomes in LMIC are due to many factors. Healthcare workers have limited skills and poor adherence to clinical guidelines; they also face large patient loads and high staff turnover, which make training difficult to implement.(3-5) Clinical decision support software (CDSS) offers features that address many of these gaps in care delivery including improving fidelity to clinical guidelines and reducing medical errors.(6-8) CDSS also has the potential to surmount training challenges. Incorporated into daily practice, CDSS offers the prospect of improvement that endures over time and across new and frequently changing staff...*
   >
   > *… the key challenge in scaling CDSS is that implementation requires initial training that has historically been done by information technology specialists or product designers, who are short in supply in low income countries. Task-*

shifting(13) of training roles and other clinical work to lower cadre health care workers has been recognized as a sustainable and cost-effective method to expand access to medical care.(14-20) This approach could provide a sustainable and successful implementation of CDSS in low income countries. However, there have been no studies examining whether tasks traditionally done by specialists related to CDSS in low-resource settings can be task-shifted to health care workers.

2. Distinguish between the two objectives. For greater clarity, we need to distinguish between 1) the acceptability of using the application and 2) the effectiveness of the fact that it is carried out by midwives. For each of these objectives, the outcomes selected to achieve them should be linked.

   Our response: We appreciate the reviewer's comment. We have distinguished the two objectives in the 'Introduction' and 'Methods' sections, as follows.

   Introduction

   Given the limited evidence for a sustainable and scalable adoption of CDSS (24-26), our aim was to evaluate whether CDSS expert roles could be task shifted to midwives in the implementation of NoviGuide. First, we developed the NoviGuide Neonatal Essentials Trainer program, a midwife-led training program for the introduction of NoviGuide in health facilities. We then applied the Kirkpatrick Model(27) to evaluate: 1) The acceptability of midwives as trainers of NoviGuide among nurse-midwives caring for newborns at four rural health facilities in eastern Uganda, and 2) The effectiveness of the training program on nurse-midwives' uptake of NoviGuide and their perception of quality of newborn care in the four rural health facilities.

   Study design

   …We used Kirkpatrick's program evaluation model. We evaluated acceptability at level 1(Reaction) for participant's reaction immediately after the initial training, and at level 3 (Behavior) for the participant's attitude towards midwives as NoviGuide Trainers at 3 and 6 months. We evaluated effectiveness at Level 2 (Learning) for participants' newly acquired knowledge and skills to use NoviGuide, and at Level 3 (Behavior) for participant's uptake of NoviGuide and perception of newborn care following the introduction of NoviGuide. (Table 1)

3. Explain why you carried out 11 focus groups?
   Our response: We now more clearly explain the selection of focus groups as follows:

   …In total, we conducted ten focus group discussions; four at the general hospital because of the large number of participants and two at each of the

three Health Center IVs. Seven to eleven participants attended each discussion.

4. You'd better explain why the 3-month and 6-month measurements answer the question. Explain why you made this choice. It's not clear, and with such a high attrition rate, how were newcomers trained?

   Our response: In this 20-month evaluation, we chose 3 and 6 months timepoints to conduct surveys and focus groups to minimize the impact of recall bias that might occur at later times points. We have included this explanation in the 'Data collection' section as follows.

   At three and six months, participants completed another survey adapted from the Electronic Health Record End User survey and participated in focus group discussions for evaluation of both effectiveness and acceptability (Kirkpatrick level 2- Learning and 3- Behavior). We chose three and six months as these timepoints were close enough to the initial training for participants to remember their perception about the program.

   To cater for the high attrition rate, we recruited all newcomers who were then trained by the NoviGuide Trainers during their monthly site support visits. We have included this detail in the text under the 'Enrollment and training of the study participants' section as follows.

   …The trainers then provided technical support for NoviGuide use and addressed any technical problems daily during the first two weeks and monthly thereafter. As an open cohort study, the trainers also trained newly recruited participants following staff changes.

5. The discussion is more like an advertising brochure than a reflection on the results. Moreover, it presents these results again without discussing them. These results should be put in perspective with what the literature says on the subject: what the literature says about the influence of making the training by healthcare professionals? in the literature, etc.?

   Our response: We appreciate the reviewer's comment. We have rewritten the 'Discussion' section incorporating the reviewer's suggestion.

Reviewer # 2

The authors evaluated the acceptability and effectiveness of midwives as trainers of a digital clinical decision-making application amongst midwives/nurses working in a selected hospital and health centers in Uganda. The study contributes to literature on shifting tasks from information technologists to health workers at primary health care. General comments:

1. Study Site selection and setting lines Page 5 27 – 43.

a. It will be useful to reader to describe study population more. What was the number of midwives in hospital and health center level? How long do they stay in their post in each facility? What is the rotation system to work in maternity/neonatal units specifically for hospital?

   Our response: We appreciate the reviewer's comment. We have included more detail about the study population in the 'Study site selection and setting' section as follows:

   > *…At the launch of the study, Tororo General Hospital had 22 midwives providing both maternal and newborn care in the labour suite, postnatal ward and a small Kangaroo Mother Care room. Staff work 2-3 per shift on each of these units with supervisory support from 2-3 medical officers. They rotate to other units at least annually. The Kangaroo Mother Care room was refurbished in September 2021 to a neonatal unit… Six staff were assigned neonatal roles to work on this unit. The three Health Center IVs… Six to eight midwives provide newborn care in addition to maternity care including labour and delivery, postnatal care and out-patients department. Staff work 1-2 per shift with supervisory support from one medical officer.*

b. The neonatal room refurbishment in September is an important detail with regards to improving quality of care for neonates in general and would impact results. This is missing in discussion and would be important to indicate if there were significant differences between 3 and 6 months for hospital level compared to health centers?

   Our response: We agree with the reviewer that the refurbishment of the neonatal room at the general hospital could have impacted the participant's perception of the effectiveness of the training program. However, during our evaluation, we did not collect data comparing the quality of care provided at different sites. We have included this detail in the 'Discussion section' as a study limitation.

   > *Our study has several limitations… One site had recently undergone refurbishment of it neonatal unit. The perception of better care resulting from new resources at that facility could have positively biased participants' perception of improved quality of newborn care.*

2. The use of the term "low resource settings" is too general for recommendations or application of this approach and suggest avoiding its use as a generality and qualifying to study/specific settings in abstract, discussion and conclusion section. The ownership of tablet ownership, internet coverage and electricity reliability are important factors that need to be considered in "cost effectiveness" or feasibility to make an overarching recommendation for low resource settings.

Our response: We agree with the reviewer that some resources need to be considered for successful implementation of CDSS in low resource settings. These resources include internet coverage, reliable electricity and ownership of mobile devices. We have revised the ‘Abstract’, ‘Discussion’ and ‘Conclusion’ sections accordingly.

3. METHODOLOGY:

   a. MAJOR COMMENT: Kirkpatrick’s four levels are 1 (reaction), 2 (learning objectives met), 3 (behavior change i.e., attitudinal or translation of learning into practice) and 4 (impact on organization, quality and user of service). Currently level 4 measure is change of behavior – which is uptake of NoviGuide and should be categorized as Level 1.
   Our response: We appreciate this comment and agree with the reviewer that uptake of NoviGuide is a measure of change of behavior- translation of learned skills into practice. We have recategorized this outcome from Kirkpatrick level 4 to level 3. This is now reflected throughout the manuscript including the abstract and Table 1.

   b. MAJOR COMMENT: Level 4 measure i.e. the uptake of use of CDSS is dependent on other factors (internet, smartphone) besides training. Not controlling for these factors makes it difficult to attribute training effectiveness. This finding and methodology to assess it is quite weak and best to limit evaluation to current levels 1, 2 and 3.
   Our response: We appreciate the reviewer’s comment and have limited our evaluation to Kirkpatrick levels 1, 2 and 3 only as explained above.

   c. What was the eligibility criterion for enrolling midwives in the facility? For instance, were all the midwives and nurses in the hospital invited to participate. What about ownership of smartphones?

      Our response: All the nurses and midwives caring for newborns at these facilities were invited to participate in the study. Other criteria included age of 18 years or more, having an active practicing license and willingness to participate in the study. We have included this detail in the ‘Enrollment and training of the study participants’ section. Ownership of a smart phone was not an eligibility criterion as the software was provided on tablets stored at the facility.

      > *All nurse-midwives at the four study sites were invited to attend an introductory session at their respective sites. Eligibility criteria included provision of newborn care at the study sites, more than 18 years of age, having an active practicing license to practice and willingness to participate in the study…*

   d. There was mention of open cohort in result section that was not covered in methods. Why was this approach used?

Our response: We appreciate this comment. We conducted an open cohort study to enroll all nurses and midwives caring for newborns in settings with high attrition rate. Staff transfers are common in these settings. Enrolling new participants following staff changes captures real world situations for NoviGuide implementation. We have revised the 'Study design' section as follows.

> *We conducted a 20-month, concurrent triangulation mixed-methods open cohort study from September 2020 to May 2022. We chose an open cohort study design to enroll all nurse-midwives following staff changes, capturing real-world situations with high attrition rates for NoviGuide implementation.*

e. Page 6 line 52. Can you explain how tablets were distributed? What was the ratio, tablet per how many nurses/midwives? Similarly, can authors explain how internet presence/absence affected use of application. About a quarter accessed internet and not clear to reader what this means, does this mean that they did not use the app?

Our response: Each site received 2 to 4 tablets depending on the number of staff on duty. We have included more information in the 'Introduction of NoviGuide at the health facilities' section as follows:

> *Each site received 2 to 4 tablets (Amazon Fire HD 8 tablet) loaded with NoviGuide. The number of tablets depended on the number of staff per shift. With 2-3 staff per shift, the general hospital received 4 tablets while the Health Center IVs each received two tablets because they have 1-2 staff per shift.*

NoviGuide works both offline and online and internet coverage did not affect uptake of NoviGuide. The software was provided on tablets and was majorly used offline. The tablets were intermittently connected to internet at least weekly to upload usage data for evaluation of uptake as described below.

> *Introduction of NoviGuide at the Health Facilities*
>
> *…The trainers identified a key contact person (NoviNurse) at each site who kept the study team informed about any challenges encountered. The NoviNurse assisted with data synchronization by intermittently connecting the tablets to WiFi provided by a MiFi modem (airtel 4G modem model MF927U).*
>
> *Data collection*
>
> *…NoviNurses connected the tablets to WiFi by switching on modems at least once every week for 20 months to transfer NoviGuide usage data to a secure cloud-based database. We collected data about the*

participant's uptake of NoviGuide to further evaluate Kirkpatrick Level 3- Behavior

At baseline, about half (46.9%) owned smart phones and a quarter assessed internet using their smart phones. In our study, uptake of NoviGuide was very high at 98%. Lack of smart phone ownership didn't affect NoviGuide use. We have included this detail in the 'Results' section as follows.

…Of 49 trained participants, 48 (98%) used NoviGuide following the training. One participant from the general hospital was transferred to another facility immediately after the training…

4. What does "sustained use" mean? (line 51 in Abstract)., best to be specific that it covered three months period.

   Our response: We appreciate the reviewer's comment. By 'sustained use', we meant that NoviGuide was used at each facility throughout the 20-month study period despite staff changes. To improve the manuscript, we have refrained from using the word 'sustained' and now use 'uptake of NoviGuide'. This change is now reflected throughout the manuscript.

5. Am curious to learn, why trainees' skills were not built on monitoring dashboards within their facilities to empower them to improve quality and not depend on trainers.

   Our response: We agree with the reviewer that empowering trainees to utilize data for their quality improvement initiatives is a potentially important use of the software. We however didn't not include this within the training program. This is an important strategy for sustainable implementation of the software that we could consider during the next evaluation. We have included this detail in the 'Discussion' section for consideration during the next evaluation as follows.

   …For sustainable use of the software, usage data could potentially be used to support quality improvement initiatives and staff supervision. Further research is therefore required to evaluate how site dashboards could be integrated into routine organizational systems.

6. Organization of manuscript – ethical considerations, funding, acknowledgement, author contribution and conflict of interest should be placed before references not in method section
   Our response: We have reorganized the manuscript as per the reviewer's suggestion.

7. Patient and public involvement lines 17 -29: The content would need to be revised. District health officer and retired midwife are professionals and do not constitute the public or patient populations. Authors would need to explain whether involvement of patients or public was appropriate or not for their study.
   Our response: We have revised the 'Patient and public involvement' section as follows.

Neither patients nor the general public were involved in the design or management of the study.

8. Result section

a. GENERAL MAJOR COMMENT: The results are biased to only positive findings which is strange given the complexity/new approach used and Table 2 indicate some negative findings on acceptability and Table 3 in use that would need to be reflected in result section and in discussion. Recommend a balanced reporting of positive and negative findings.

Our response: We appreciate the reviewer's comment. While two questions in the survey (Table 2) about the reaction to the training aimed to solicit negative consequences, participants' replies indicated none. So, we effectively had no negative or critical responses. While this could be interpreted as success, it also raises the possibility of positive reporting biases that we acknowledged as a limitation in the 'Discussion' section as follows.

> *Our study has several limitations... While the universally positive opinions from participants about the program is encouraging, it raises concerns about reporting bias; it is possible that participants felt obligated to give their supervisors positive reviews as trainers.*

b. It would be nice to see more demographic details on study population such as the mean (range) time working within maternity/neonatal units, professional years of work in general

Our response: We have included more demographic details in the 'Results' section as follows.

> *We screened 49 female nurse-midwives…. Of 49 participants, 21 (42.7%) had work experience of 3-10 years, 12(24.5%) had worked for 11-20 years, 11 (22.5%) for 0-2 years and only 5 (10.2%) for more than 21 years.*

c. Results on drug dosing are hard to interpret. What is the intention of communicating these results. If it is to describe the commonest areas used in NoviGuide, suggest to enter the findings in a table indicating what aspect of dosage was often accessed, admissions, discharge etc. disaggregating by health facility type (hospital vs health center level). What does the percentage mean? What is the denominator?

Our response: To improve the clarity of 'Results' section on uptake of NoviGuide, we have decided to delete the drug dosing results and it now reads as follows:

> *The usage data aligned with the focus group discussion data about the participant's use of NoviGuide…In 20 months, from September 2020 to*

May 2022, a total of 4045 assessments of newborns were entered into NoviGuide…

The participants used NoviGuide for varying newborn conditions. Of all the assessments made into NoviGuide, 13.8% (558/4045) were for preterm newborns, 17.5% (709/4045) for newborns weighing under 2.5kg, and 21.1% (855/4045) had a temperature less than 36.5°C.

9. Discussion
   a. The first two paragraphs is a repeat of study findings and could be summarized into two lines.
   Our response: We appreciate the reviewer's comment and have rewritten the first paragraph as follows.

   To our knowledge, this is the first study evaluating a program that task-shifts CDSS expert roles to midwives in the introduction of a complex neonatal CDSS platform in rural health facilities. The participants revealed a high degree of acceptance of midwives as NoviGuide Trainers. The midwife-led training program resulted in high uptake of NoviGuide among participants and perceived improvement in newborn care practices.

   b. Recommend section to be written to focus on result interpretation, for instance, what needs to be studied further.
      i. For instance, questions that remain unanswered is the effectiveness of midwives trainers on the quality of neonatal care (level 4) or long term impact on uptake given that assessment was 3 – 6 months post intervention which is short and Hawthorne effect/bias could have been high
      ii. What does this scale up of this approach at subnational or national level and its implications in selection and training of trainers? Would more research need to be done on how monitoring could be integrated within routine supervision or more investigation made on time spent in troubleshooting and the appropriate number of trainers to cover health facilities

   Our response: We appreciate the reviewer's suggestions. From our 20-month long evaluation of the program, we agree that there still remain unanswered questions on the effectiveness of the training program on the quality of newborn care and long-term use of the software at the facility level. We have rewritten the 'Discussion' section as per the reviewer's suggestions including details for consideration during large scale implementation of the program.

c. What does turnover of staff mean, mobile ownership, internet usage, how could this affect overall effectiveness? Can this be controlled in future studies? Have other studies looked at this?
Our response: We appreciate the reviewer's comment. Turnover of staff means the rate at which staff are rotated within health facilities. Staff transfers are very common in these settings and could present implementation challenges at the facility level. We conducted an open cohort study to enroll all the nurse-midwives and exemplify real world situations for NoviGuide implementation. Contrary to other studies, smart phone ownership did not affect use of the software. The uptake of NoviGuide was very high at 98% in our study. We agree with the reviewer that availability of internet needs consideration for large scale implementation. In our study, the software was downloaded onto tablets and use was majorly offline. We have included all this detail in the 'Discussion' section as per the reviewer's suggestion.

VERSION 2 - REVIEW

Reviewer 1

Name Abt, Maryline

Affiliation University of Applied Sciences Western Switzerland La Source Institute and School of Nursing

Date 24-Jan-2024

COI I declare no conflict of interest

Corrections are acceptable for publication

Reviewer 3

Name Kurth, Tobias

Affiliation Charité Universitätsmedizin Berlin, Institute of Public Health

Date 18-Jul-2024

COI TK has received research grants from the German Federal Joint Committee (G-BA). He also received personal compensation from Eli Lilly & Company, Novartis, the BMJ, and Frontiers.

In this mixed methods study, Muhindu and colleagues aimed to evaluate the acceptability and effectiveness of midwives as trainers for NoviGuide, a neonatal clinical decision support

system (CDSS). Forty-nine nurses were included, and a midwife-led trainer program was implemented in three three-hour sessions. Surveys and focus groups at baseline, 3 and 6 months were used, and usage data was viewed from September 2020 through May 2022. Results indicated that the midwife-led program was acceptable and led to self-reported improvement in newborn care and high uptake of NoviGuide among nurse-midwives.

The study was well-designed and conducted. The reporting is transparent, and the conclusions reflect the results provided. This is a revised version; the authors responded in great detail to the prior reviews. I agree with the revisions. However, I do have a few more points for the authors to consider.

1. In the Strength and Limitations section, the authors speak of “robust” multi-site, mixed-methods evaluation. As the word robust is unclear, I suggest omitting it.

2. In the Data Analysis section, the authors report the equation they used for the effect estimated (r=Z/N). However, what the letters stand for needs to be clarified. I assume that r is the effect size, Z is the z-statistic, and N is the total sample size. If yes, I recommend using lowercase and italicized for all letters. Is the equation to calculate effect sizes for a Wilcoxon Signed-Rank Test r = z / sqrt(n) (e.g., https://search.r-project.org/CRAN/refmans/rstatix/html/wilcox_effsize.html)? This would result in an effect size between 0 and 1, which would also go along with the chosen cutoffs for the effect sizes of 0.2 to <0.5 as small, 0.5 to <0.8 as medium, and 0.8 and above as large.

3. The authors report to have used Microsoft Excel to calculate the effect sizes. The rest of the quantitative data analyses were done using SATA. Why not use STATA to estimate the effect size? I recommend also showing the 95% confidence intervals for the effect sizes. How was the z-statistic calculated in Microsoft Excel?

4. I suggest removing the sentence that this is the first study evaluating a program that task-shifts CDSS expert roles to midwives, as this is not a quality or scientific statement.

5. The authors should discuss possible selection bias and discuss the potential implications of this bias for the generalizability of the findings.

6. Can the authors provide the grand number that funded the research?

7. The reporting of P values should follow common reporting guidelines (P >0.1 with two decimal places, between 0.001 and 0.1 with three, and below 0.001 as <0.001).

VERSION 2 - AUTHOR RESPONSE

Reviewer #3

1. In the Strength and Limitations section, the authors speak of “robust” multi-site, mixed-methods evaluation. As the word robust is unclear, I suggest omitting it.

Our response: We have omitted the word ‘robust’ from the first sentence as follows:

A multi-site, mixed-methods evaluation using Kirkpatrick Model (level 1-3).

2. In the Data Analysis section, the authors report the equation they used for the effect estimated (r=Z/N). However, what the letters stand for needs to be clarified. I assume that r is the effect size, Z is the z-statistic, and N is the total sample size. If yes, I recommend using lowercase and italicized for all letters. Is the equation to calculate effect sizes for a Wilcoxon Signed-Rank Test r = z / sqrt(n) (e.g., https://search.r-project.org/CRAN/refmans/rstatix/html/wilcox_effsize.html)? This would result in an effect size between 0 and 1, which would also go along with the chosen cutoffs for the effect sizes of 0.2 to <0.5 as small, 0.5 to <0.8 as medium, and 0.8 and above as large.

   Our response: We appreciate the reviewer’s comment. In response to this comment (and the next), we now use Cohen’s d test to compute the effect sizes (in STATA), and have revised the Data Analysis section accordingly.

   …We then used a two-tailed Wilcoxon signed-rank test to compare the median scores at 3 and 6 months. To determine the magnitude of the differences, we calculated effect size using Cohen’s d test. A positive effect size indicated an increase in the median score while a negative effect size indicated a decrease. We considered effect sizes of 0.2 to <0.5 as small, 0.5 to <0.8 as medium and 0.8 and above as large (31) …We analyzed all quantitative data using Stata 16 (StataCorp, College Station, Texas) setting the confidence interval at 95% and considered P value of <0.05 significant.

3. The authors report to have used Microsoft Excel to calculate the effect sizes. The rest of the quantitative data analyses were done using SATA. Why not use STATA to estimate the effect size? I recommend also showing the 95% confidence intervals for the effect sizes. How was the z-statistic calculated in Microsoft Excel?

   Our response: We now use STATA to calculate the effect size. As per the reviewer’s comment, we have also revised Table 4 showing both the Wilcoxon sign-rank test and Cohen’s d test results including the 95% confidence intervals for the effect sizes.

4. I suggest removing the sentence that this is the first study evaluating a program that task-shifts CDSS expert roles to midwives, as this is not a quality or scientific statement.
   Our response: As per the reviewer’s comment, we have revised the first paragraph as follows:

In our study, the participants revealed a high degree of acceptance of midwives as NoviGuide Trainers. The midwife-led training program resulted in high uptake of NoviGuide among participants and perceived improvement in newborn care practices.

5. The authors should discuss possible selection bias and discuss the potential implications of this bias for the generalizability of the findings.
   Our response: We appreciate this suggestion, and have more clearly described a potential impact of selection bias in our limitations paragraph, as follows:

   To avoid selection bias, we enrolled all nurses and midwives caring for newborns at the respective health facilities. However, two trainers in our program had previous experience with NoviGuide; this likely contributed to the effectiveness of our training program.

6. Can the authors provide the grant number that funded the research?
   Our response: We have included the grant number as follows:

   This project was supported by East Africa Preterm Birth Initiative generously funded by Bill & Melinda Gates Foundation (Grant number: OPP1107312).

7. The reporting of P values should follow common reporting guidelines (P >0.1 with two decimal places, between 0.001 and 0.1 with three, and below 0.001 as <0.001).

   Our response: We have revised Table 4 as per the reviewer's comment.

VERSION 3 - REVIEW

Reviewer **3**

Name **Kurth, Tobias**

Affiliation **Charité Universitätsmedizin Berlin, Institute of Public Health**

Date **01-Sep-2024**

COI **TK has received research grants from the German Federal Joint Committee (G-BA). He also received personal compensation from Eli Lilly & Company, Novartis, the BMJ, and Frontiers.**

I would like to thank the authors for their detailed responses. While the manuscript has improved, a few things remain that the authors may want to consider.

1. The authors now calculated Cohen's d as an effect size estimate. Please note that this is not a "test" so just refer to it as Cohen's d. Based on the provided reference (31 in the revised paper), I assume that the authors calculated Cohen's d as the difference of the two means divided by the pooled standard deviation. Please confirm. While Cohen's d can take negative values, the authors may want to make clear that the cutoffs of the effect sizes hold for positive and negative values, as it may otherwise be confusing.

2. The authors inconsistently cite in the text (i.e., citation after a period or comma or before it). This should be unified based on BMJ Open style.

VERSION 3 - AUTHOR RESPONSE

Responses to reviewer's comments for manuscript titled 'Midwives as trainers for a neonatal clinical decision support system at four rural health facilities in eastern Uganda: A mixed methods observational study'.

Reviewer #3

1. The authors now calculated Cohen's d as an effect size estimate. Please note that this is not a "test" so just refer to it as Cohen's d. Based on the provided reference (31 in the revised paper), I assume that the authors calculated Cohen's d as the difference of the two means divided by the pooled standard deviation. Please confirm. While Cohen's d can take negative values, the authors may want to make clear that the cutoffs of the effect sizes hold for positive and negative values, as it may otherwise be confusing.

   Our response: We appreciate the reviewer's comment. We confirm that we calculated Cohen's d as the difference of the two means divided by the pooled standard deviation, using Stata. We have revised the Data Analysis section accordingly.

   > *…To determine the magnitude of the differences, we calculated effect size using Cohen's d. A positive effect size indicated an increase in the mean score while a negative effect size indicated a decrease. We considered effect sizes of 0.2 to <0.5 as small, 0.5 to <0.8 as medium and 0.8 and above as large (31).*

2. The authors inconsistently cite in the text (i.e., citation after a period or comma or before it). This should be unified based on BMJ Open style.

   Our response: We have reviewed the whole manuscript and unified the citations based on BMJ Open style.
